# Few-Cycle Surface Plasmon Polaritons

**DOI:** 10.1021/acs.nanolett.3c04991

**Published:** 2024-02-12

**Authors:** Kazma Komatsu, Zsuzsanna Pápa, Thomas Jauk, Felix Bernecker, Lázár Tóth, Florian Lackner, Wolfgang E. Ernst, Harald Ditlbacher, Joachim R. Krenn, Marcus Ossiander, Péter Dombi, Martin Schultze

**Affiliations:** † Institute of Experimental Physics, Graz University of Technology, 8010 Graz, Austria; ‡ Wigner Research Centre for Physics, 1121 Budapest, Hungary; § ELI-ALPS Research Institute, 6728 Szeged, Hungary; ∥ Institute of Physics, University of Graz, 8010 Graz, Austria; ⊥ Harvard John A. Paulson School of Engineering and Applied Sciences, Harvard University, Cambridge, Massachusetts 02138, United States

**Keywords:** surface plasmon polaritons, ultrafast plasmonics, plasmonic waveguides, femtosecond dynamics

## Abstract

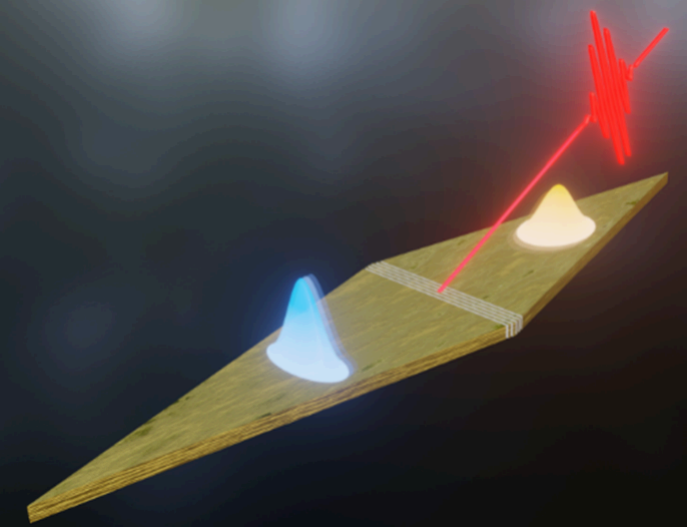

Surface plasmon polaritons (SPPs) can confine and guide light in nanometer volumes and are ideal tools for achieving electric field enhancement and the construction of nanophotonic circuitry. The realization of the highest field strengths and fastest switching requires confinement also in the temporal domain. Here, we demonstrate a tapered plasmonic waveguide with an optimized grating structure that supports few-cycle surface plasmon polaritons with >70 THz bandwidth while achieving >50% light-field-to-plasmon coupling efficiency. This enables us to observe the—to our knowledge—shortest reported SPP wavepackets. Using time-resolved photoelectron microscopy with suboptical-wavelength spatial and sub-10 fs temporal resolution, we provide full spatiotemporal imaging of co- and counter-propagating few-cycle SPP wavepackets along tapered plasmonic waveguides. By comparing their propagation, we track the evolution of the laser-plasmon phase, which can be controlled via the coupling conditions.

Controlling electric fields on a subwavelength scale with petahertz clock rates has emerged as one of the key challenges for future information processing in optoelectronic devices. Surface plasmon polaritons (SPPs)^1−4^ proved to be formidable candidates in photonic circuits as they allow for steering and concentrating light below the diffraction limit.^5−8^ Merging SPPs with few-cycle light bursts furthermore enables to confine them not only in space but also in time, creating field strengths capable of driving nonlinear processes even at input laser pulse energies too low to induce a nonlinear response.^9−12^ The generation of few-cycle SPP wavepackets, however, requires an efficient coupling scheme for broad-bandwidth light. Grating couplers compensate for the photon-plasmon momentum mismatch^7,13^ and have been utilized to excite SPPs in various configurations, such as grooves,^14−21^ protrusions^17,22^ or arrays.^23,24^ In this work, we develop full spatiotemporal imaging of ultrashort plasmonic wavepackets and demonstrate the generation of the shortest few-cycle SPP wavepackets to date with the help of broadband grating couplers (for an overview of experimentally achieved SPP durations, see Table 1).

**Table 1:** Temporal Duration of Co-/Counter-Propagating SPP Wavepackets

	Paper	Laser pulse length (fs)	SPP wavepacket (fs)	Propagation distance of SPP (μm)	Sample material
co-propagation	Vogelsang et al., 2015^15^	16	27	40–50	gold nanotip
	Gong et al., 2015^16^	15	28	135	thin gold film
	Kahl et al., 2018^18^	15	16	40	thin gold film
	Kravtsov et al., 2013^19^	10	20	20	gold nanotip
	Berweger et al., 2011^20^	10	16	20	gold nanotip
	Yi et al., 2017^21^	5	11	40	thin gold film
	**this work**	**7.6**	**7.9**	**40**	**thin gold rhombus**
counter-propagation	Crampton et al., 2019^23^	15	>50	74	thin silver film
	Crampton et al., 2022^24^	15	25–50	15–20	thin silver film
	Lemke et al., 2012^17^	15	20	18	thin gold film
	**this work**	**7.6**	**17.6**	**40**	**thin gold rhombus**

The momentum of an SPP  depends on its frequency ω, the speed of light in vacuum *c*, and the permittivity ε of the material that it propagates in. Grating couplers function by providing a momentum  inversely proportional to their grating period Λ. When light (with momentum *k*_*L*_) is normal-incident on a grating coupler (see Figure 1(a) for a schematic), it can generate plasmons if the grating momentum is equal to the SPP momentum *k*_SPP_, because the momentum of light *k*_*L*__,||_ projected on the plasmon propagation is zero.

**Figure 1:**
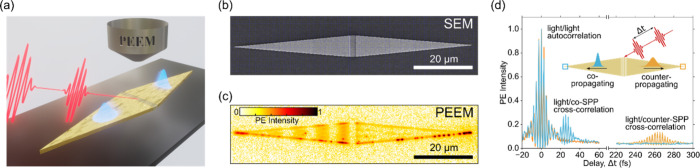
(a) Experimental scheme. By employing few-cycle light pulses, SPP wavepackets are generated with a grating coupler consisting of five grooves milled into the middle of a rhombic gold structure. Owing to the different coupling conditions, the co- and counter-propagating plasmonic waveforms have different spectral bandwidths and duration. Photoemission electron microscopy (PEEM) thereby allows for capturing the spatiotemporal evolution of the plasmonic wavepackets on the way to the apices. (b), (c) Images of the rhombic gold waveguide recorded by (b) scanning electron microscopy (SEM) and (c) PEEM. (d) Time-resolved photoemission yield extracted from the apices. The experiment is an interferometric autocorrelation measurement with three pulses, resulting in a light/light autocorrelation and the light/SPP cross-correlations that provide pulse durations of 7.6 fs (light), 7.9 fs (co-propagating SPP), and 17.6 fs (counter-propagating SPP), respectively.

For light incident at an angle ϑ to the surface normal, *k*_*L*__,||_ = *k*_*L*_ sin(ϑ) is nonvanishing, and a grating coupler can launch SPPs that propagate along *k*_*L*__,||_ (co-propagating) and SPPs that propagate antiparallel to *k*_*L*__,||_ (counter-propagating).^25^ When *k*_SPP_^co-prop^/*k*_SPP_^counter-prop^ are the momenta of the co/counter-propagating plasmon, the oblique incidence plasmon excitation condition is described^26^ by
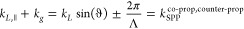
1For a grating coupler with limited spatial extent, the number of grooves plays a decisive role for both the spectral coverage and the efficiency.^27^ A single slit acts as a perfect bandwidth transmitter; however, it suffers from poor coupling efficiency. Inversely, many slits increase the efficiency but narrow the bandwidth.

In order to achieve few-cycle SPPs, we designed a grating coupler integrated in a 100-nm-thick gold film by using finite-difference time-domain (FDTD) simulations and adjusting the number of grooves, the duty cycle, and the periodicity (see Figures S2–S4 in the Supporting Information for details). The optimal grating period was found to be 390 nm with a 50% duty cycle. Light that illuminates the grating coupler at a 65° angle of incidence with respect to the surface normal and is *p*-polarized can launch co-propagating and counter-propagating SPPs; see Figure 1(a). The coupler supports SPP wavepackets with 76 THz spectral bandwidth in the co-propagating direction and with 31 THz spectral bandwidth in the counter-propagating direction and a peak coupling efficiency of more than 50% for both propagation directions.

We fabricated an 80-μm-long gold rhombic waveguide on a silicon substrate via electron beam lithography and subsequent lift-off and patterned the grating coupler via focused ion beam milling. Figure 1(b) shows a scanning electron microscopy picture of the sample. To explore its ultrafast properties, we use a few-cycle light pulse (7.6 fs temporal duration) to launch plasmonic wavepackets that propagate toward each apex. After a delay time Δ*t*, we interfere a replica of the pump pulse with the SPPs and record the spatially resolved photoelectron emission in a photoelectron emission microscope (PEEM). The two-dimensional map of photoemission consists of photoelectrons created by each individual light pulse, the interferometric autocorrelation (coherent artifact) of both pulses’ fields, and the cross-correlation between the plasmon field created by one laser pulse and the other laser pulse’s light field. The latter two depend on Δ*t*, but, beyond the coherence time of the laser pulses, only the cross-correlation signal depends on Δ*t*; thus, the measurement scheme enables one to spatiotemporally track the evolution of the plasmonic wavepackets. Given the non-normal incidence of the light field on the gold structures, we can distinguish between co- and counter-propagating plasmonic waveforms (see Figure 1(c) for a PEEM image). The interference between the SPP wave and the light field launching the SPP results in the formation of Moiré patterns, which vary with respect to the edge orientation of the rhombus.^28,29^

In our measurement, the carrier envelope phase (CEP) of the laser pulse is not actively stabilized. However, the optical response leading to the formation of the plasmonic wavepacket is linear, and thus, the CEP of a plasmon wavepacket is rigidly linked to that of the exciting laser field. As a result, the correlation traces depending on the absolute value of the interference field |*E*_*int*_(*t;τ*)| at time *t* and delay time τ are insensitive to the CEP of the exciting laser pulse *φ*_*L,CEP*_:
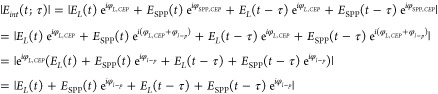
2Here *E*_*L*_(*t*) (*E*_SPP_(*t*)) represents the field amplitude of the laser (the SPP wavepacket) when the laser’s CEP φ_*L,CEP*_ and the SPP’s CEP φ_SPP,*CEP*_ = φ_*L,CEP*_ + φ_*l–p*_ are zero. The above equation validates that the properties of the correlation traces, namely, the intensity and the phase, are independent of the laser’s CEP. The SPP’s CEP contains the laser-plasmon phase φ_*l–p*_, which is the phase difference between the oscillating laser and plasmon field, and the phase difference between the SPP and the laser is independent of the laser’s CEP.

Figure 1(d) shows the time-resolved photoemission yields extracted from the apices. At zero delay, we observe the light/light third-order interferometric autocorrelation, yielding a temporal duration of 7.6 fs for the laser pulses.^30^ The third-order nonlinearity comes from the nature of the photoemission process, which is determined by the ratio of the work function (4.8 eV^31^) to the light photon energy (1.6 eV), and the power dependence of photoelectron counts also supports the third-order photoemission (see Figure S1 in Supporting Information). When the SPP wavepackets reach the apices, they modulate the photoemission yield based on the SPP/light third-order cross-correlation, which provides pulse durations of around 7.9 and 17.6 fs for the co- and counter-propagating SPP wavepackets, respectively.^30^ In the case of co-propagation, the grating couples most of the bandwidth of the femtosecond pulse into the plasmonic wavepacket and the SPP wave suffers little dispersion upon traveling over 40 μm, resulting in a short SPP pulse (see Table 1). As expected from the design, in the counter-propagating direction, the grating coupler launches a plasmon with a narrower bandwidth and thus longer duration.

The spatiotemporal evolution of the SPP wavepackets along the lower edge of the rhombic gold waveguide is depicted in Figure 2(a). The center of the grating is located at position 0 μm, negative values correspond to co-propagation, and positive values correspond to counter-propagation. Their slopes show a clear difference in the space-time landscape that can be reproduced by FDTD simulations (Figure 2(b)) considering the experimental geometry (for details, see Methods).

**Figure 2:**
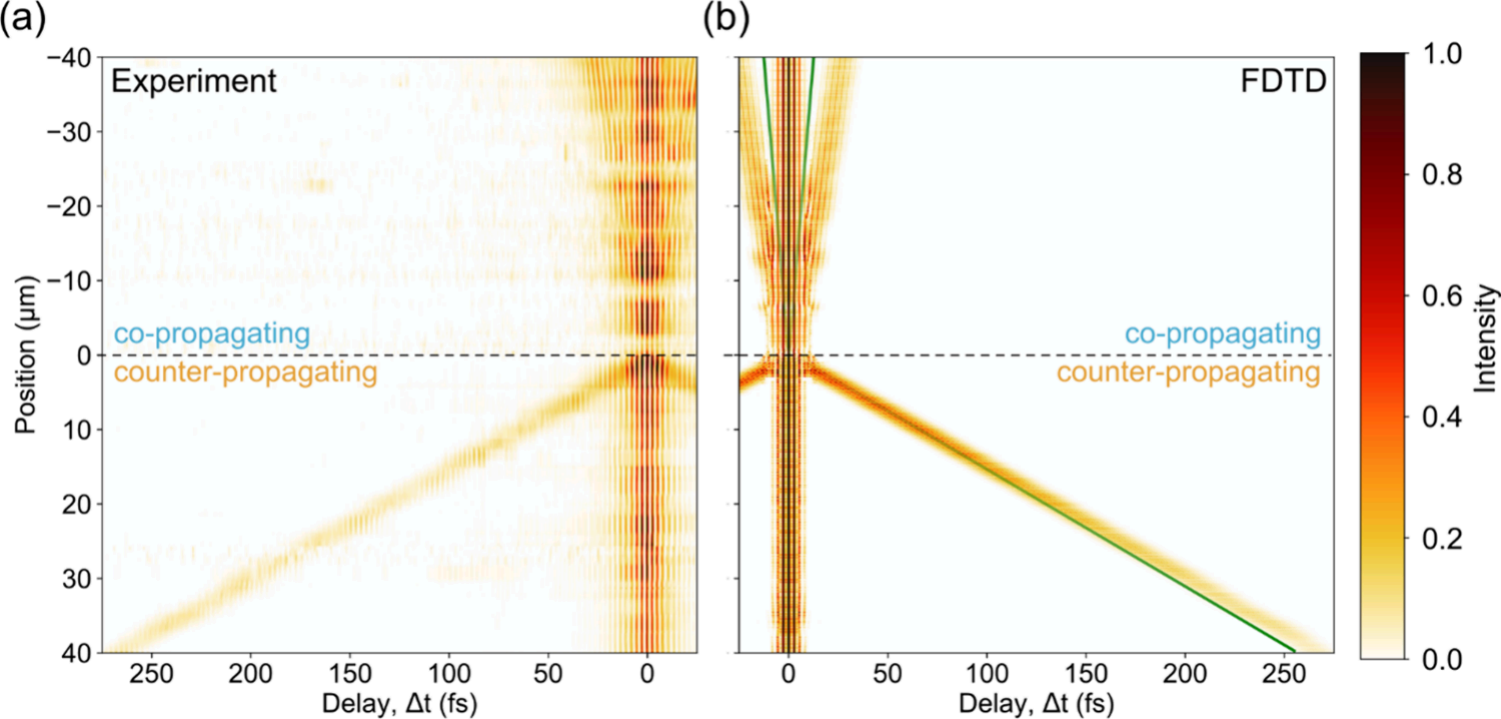
Spatiotemporal evolution of the SPP wavepackets. (a) Time-resolved photoemission yield extracted along the lower edge of the rhombic gold structure. Grating position: 0 μm; apices: ±40 μm. (b) Photoemission intensity modeled by finite-difference time-domain (FDTD) simulation of the experimental geometry (according to eq 4). Green lines in panel (b) denote the space-time evolution of a hypothetical SPP traveling with a group velocity equal to the speed of light in a vacuum (light-line, *v*_*g*__,∓_ = *c* in eq 3).

The difference in delay time is explained by the oblique incidence of the light pulses,^17,23^ which makes the experiment probe the SPPs’ group velocities relative to the in-plane component of the speed of the probe light pulses. The group velocities of the plasmonic wavepackets can be deduced by fitting the envelope of the space-time evolution with
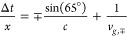
3where *x* is the position of the SPPs with respect to the grating coupler, Δ*t* is the delay time between the laser pulses, *c* is the speed of light and *v*_*g*__,∓_ is the group velocity of the co- and counter-propagating SPPs. This results in *v*_*g*__,–_ = *v*_*g,*__co-prop_ = (0.91 ± 0.02)*c* and *v*_*g*__,+_ = *v*_*g,*__counter-prop_ = (0.93 ± 0.02)*c*. Figure 3(a) compares the experimentally extracted values with the frequency-resolved group velocity predicted from the optical properties of gold,^32^ which coincide with previous studies.^17,18,33^

**Figure 3:**
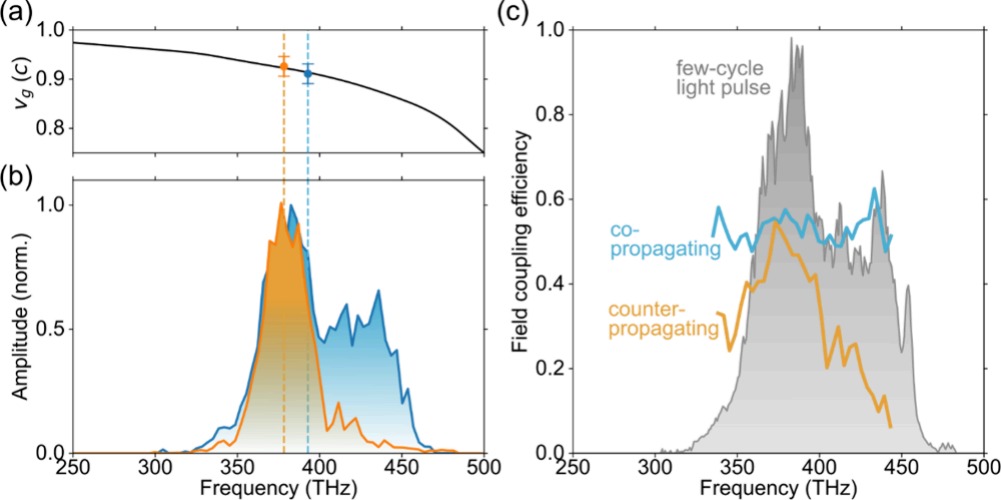
Spectra and field coupling efficiencies for the co-propagating (blue) and counter-propagating (orange) SPP wavepackets. (a) Frequency-dependent group velocity *v*_*g*_ of SPP wavepackets on a gold surface. The black line corresponds to the analytical calculation, and the blue/orange dots correspond to the values deduced from the experiment. (b) Spectra of the co- and counter-propagating SPP wavepackets calculated by Fourier transforming the cross-correlations at each apex. (c) Extracted field coupling efficiencies of the observed plasmonic waveforms compared to the spectrum of the light pulse (gray shaded area), which is centered at 387 THz.

The cross-correlation signals at the apices permit extraction of the SPP spectra, as shown in Figure 3(b). While in the case of co-propagation the almost complete light spectrum is transmitted to the plasmonic wavepacket, the counter-propagating SPP’s bandwidth is distinctly narrowed, and its center frequency is red-shifted by 15 THz. Figure 3(c) presents the frequency dependent field coupling efficiencies *η*^*field*^ (ω) with respect to the laser spectrum (detailed derivation of *η*^*field*^ (ω) is provided in section 5 of Supporting Information). In good agreement with our design, the co-propagating geometry possesses a relatively uniform efficiency (50–60%) over the entire laser pulse spectrum, whereas the counter-propagating SPP’s coupling efficiency, in general, is lower and drops at frequencies beyond 400 THz.

The measurement also allows tracking of the phase evolution upon propagation of the plasmonic wavepackets with respect to the laser pulse that launches the SPPs. Experimental data are summarized in Figure 4(a), (b). The phase evolution is composed of an initial phase offset and a propagation phase that the plasmonic waveforms acquire as they travel along the rhombic gold structure. The latter increases linearly with the plasmon’s propagation distance.

**Figure 4:**
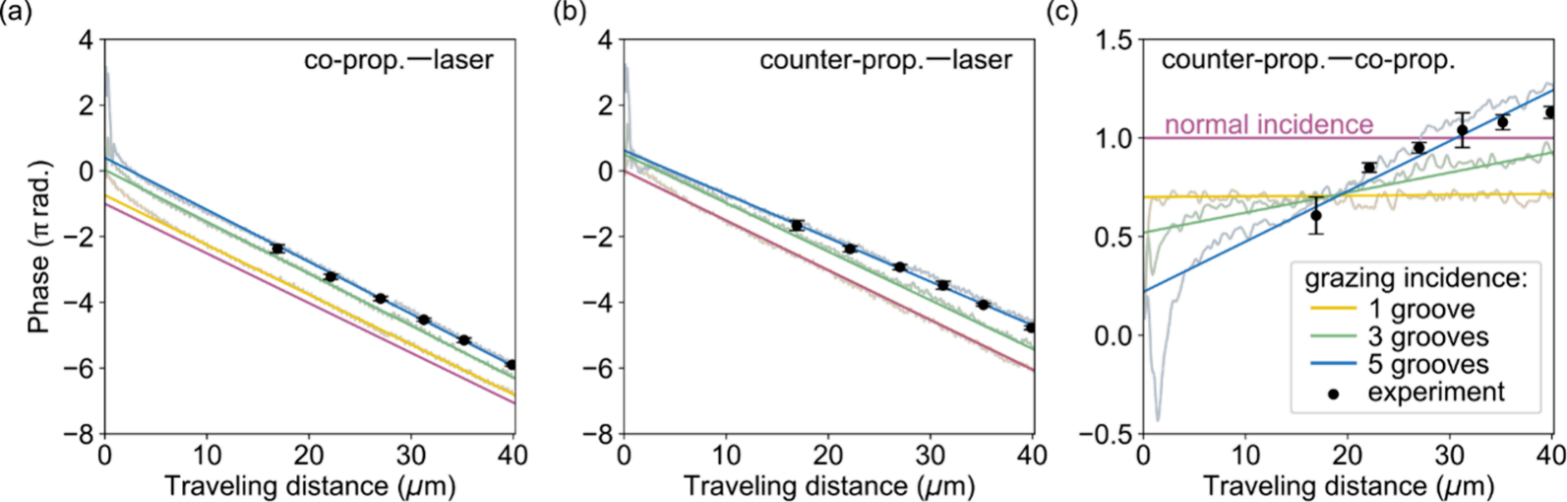
Phase evolution of the SPP wavepackets. Laser-plasmon phase of (a) the co-propagating (φ_*l–p*_^co^) and (b) the counter-propagating SPP (φ_*l–p*_^counter^). The experimental phase slip between the laser and plasmon field along the gold rhombus (black dots) is compared to a set of analytical calculations (colored lines, legend in panel (c)), assuming grazing and normal incidence, and to FDTD simulations (gray lines). (c) Relative phase φ_*l–p*_^counter^ – φ_*l–p*_^co^ between the co-propagating SPP and the counter-propagating SPP. The error bars are determined using the bootstrap method (see Methods).

To explore the components of the phase evolution further, we performed FDTD simulations for grating couplers consisting of five grooves and additionally one and three grooves and a coupler illuminated under normal incidence. The modeling results match the experimental data well. For normally incident light, the coupling phase difference between the co- and counter-propagating SPP is π resembling the bipolar oscillation of the exciting light field, and both SPP wavepackets accumulate propagation phase at the same rate, which is reflected in their constant relative phase shown in Figure 4(c). Oblique light incidence introduces a coupling phase difference considerably different from π, which decreases with the number of grooves in the grating coupler. Both experimental and modeled data reveal that the counter-propagating SPP acquires a laser-plasmon phase at a smaller rate than the co-propagating SPP.

For the interpretation of the phase evolution, we consider an analytical approach based on the group and phase velocities (details in section 7 of the Supporting Information). This estimation (see Figure 4, colored lines) retrieves the same slopes as the experiment and the FDTD simulation, suggesting that the difference in spectral composition of the SPPs causes phase slip upon propagation. The different starting offset of the laser-plasmon phase directly at the grating can be attributed to their phase mismatch inside the grating^34,35^ and the interference between the dipoles created within each grating period owing to the oblique incidence.

In summary, we demonstrated the generation of few-cycle sub-10 fs SPP wavepackets with full spatiotemporal imaging of the wavepacket evolution along a plasmonic taper with high spatiotemporal resolution. Equipping a rhombic gold waveguide with a broadband grating coupler enabled the spatiotemporal tracking of co- and counter-propagating plasmonic wavepackets in a pump–probe photoemission experiment. The experiment allowed us to fully characterize the temporal, spectral, and phase evolutions of the dispersing SPPs. Both the properties of the used grating coupler and the incidence angle determine the center-frequency and the bandwidth of the coupled SPP wavepackets. Even shorter SPPs with controlled field geometry and enhanced field asymmetries to drive nonlinear effects can be synthesized in future experiments using additional synthesizer channels, which would allow further enhancement of the cumulative bandwidth of the synthesized SPP wavepacket. This idea is in analogy with femtosecond pulse synthesis where 3 or 4 channels of an interferometer were used for free-space femtosecond pulses having different central frequencies in order to synthesize an optical attosecond pulse.^36,37^ The surface-integrated version of such an interferometric scheme would transfer this concept to the realm of plasmonics.

## Methods

The photoemission experiments were performed by using an ultrafast mode-locked Ti:sapphire laser oscillator (Rainbow, Femtolasers) with a repetition rate of 78 MHz and a pulse energy of 3 nJ. The broadband laser pulses were compressed to 7.6 fs by using a set of dispersion compensating chirped mirrors. A Mach–Zehnder interferometer was used to introduce a controlled delay between the pump and probe pulses. The *p*-polarized laser pulses were focused onto the sample with an angle of 65° with respect to the surface normal, resulting in a focal spot size of approximately (200 × 100) μm. The few-cycle pulses triggered a three-photon-photoemission process at the gold surface (Figure S1 in the Supporting Information). The two-dimensional photoelectron distribution was captured by a photoemission electron microscope (nanoESCA,^38^ FOCUS and Scienta Omicron).

We performed 2D-FDTD simulations of a gold thin film (thickness 100 nm) on a semi-infinite slab of silicon using a commercial package (Lumerical FDTD solutions, Ansys Inc.). In the center of the structure, slits with a 195 nm width were removed. For gold we use the optical constants from Johnson and Christy,^32^ and for silicon we use the optical constants from Palik.^39^ The sample was tilted by 65° to account for oblique light incidence. The following light pulse parameters were used: Gaussian spatial profile with a 20 μm waist, 375 THz center frequency, 10 fs fwhm duration, and *p*-polarization. Simulations were run using a 10 nm uniform mesh, and position-dependent SPP fields were recorded at 7.5 nm distance above the gold surface. After the simulations, assuming a three-photon process, we estimated the photoemission intensity *I*(*x,τ*) at propagation distance *x* and delay time τ using the simulated electric field of the light pulse *E*_*L*_, the SPP field *E*_SPP_, and
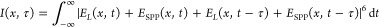
4We estimated the errors of the laser-plasmon phases using the bootstrap method.^40^ First, we randomly specified the spatial window size and obtained correlation signals from the photoelectron images in the selected spatial window. Afterward, we evaluated the laser-plasmon phases and iterated the above procedure three times to evaluate the errors. The error of the relative phase was calculated by the propagation of uncertainty.

## Supporting Information Available

Additional information on power dependence of photoelectron counts, grating coupler modeling, and other formulations used in the paper (PDF)

## References

[ref1] Barnes W. L.; Dereux A.; Ebbesen T. W. Surface Plasmon Subwavelength Optics. Nature 2003, 424 (6950), 824–830. 10.1038/nature01937. 12917696

[ref2] Ozbay E. Plasmonics: Merging Photonics and Electronics at Nanoscale Dimensions. Science 2006, 311 (5758), 189–193. 10.1126/science.1114849. 16410515

[ref3] Dabrowski M.; Dai Y.; Petek H. Ultrafast Photoemission Electron Microscopy: Imaging Plasmons in Space and Time. Chem. Rev. 2020, 120 (13), 6247–6287. 10.1021/acs.chemrev.0c00146. 32530607

[ref4] Dombi P.; Pápa Z.; Vogelsang J.; Yalunin S. V.; Sivis M.; Herink G.; Schäfer S.; Groß P.; Ropers C.; Lienau C. Strong-Field Nano-Optics. Rev. Mod. Phys. 2020, 92 (2), 025003 10.1103/RevModPhys.92.025003.

[ref5] Engheta N. Circuits with Light at Nanoscales: Optical Nanocircuits Inspired by Metamaterials. Science 2007, 317 (5845), 1698–1702. 10.1126/science.1133268. 17885123

[ref6] MacDonald K. F.; Sámson Z. L.; Stockman M. I.; Zheludev N. I. Ultrafast Active Plasmonics. Nature Photon 2009, 3 (1), 55–58. 10.1038/nphoton.2008.249.

[ref7] Smirnov V.; Stephan S.; Westphal M.; Emmrich D.; Beyer A.; Gölzhäuser A.; Lienau C.; Silies M. Transmitting Surface Plasmon Polaritons across Nanometer-Sized Gaps by Optical near-Field Coupling. ACS Photonics 2021, 8 (3), 832–840. 10.1021/acsphotonics.0c01797.

[ref8] Rácz P.; Pápa Z.; Márton I.; Budai J.; Wróbel P.; Stefaniuk T.; Prietl C.; Krenn J. R.; Dombi P. Measurement of Nanoplasmonic Field Enhancement with Ultrafast Photoemission. Nano Lett. 2017, 17 (2), 1181–1186. 10.1021/acs.nanolett.6b04893. 28094992

[ref9] Pisani F.; Fedeli L.; Macchi A. Few-Cycle Surface Plasmon Polariton Generation by Rotating Wavefront Pulses. ACS Photonics 2018, 5 (3), 1068–1073. 10.1021/acsphotonics.7b01347.

[ref10] Dombi P.; Irvine S. E.; Rácz P.; Lenner M.; Kroó N.; Farkas G.; Mitrofanov A.; Baltuška A.; Fuji T.; Krausz F.; Elezzabi A. Y. Observation of Few-Cycle, Strong-Field Phenomena in Surface Plasmon Fields. Opt. Express 2010, 18 (23), 24206–24212. 10.1364/OE.18.024206. 21164766

[ref11] Földi P.; Márton I.; Német N.; Ayadi V.; Dombi P. Few-Cycle Plasmon Oscillations Controlling Photoemission from Metal Nanoparticles. Appl. Phys. Lett. 2015, 106 (1), 013111 10.1063/1.4905464.

[ref12] Dombi P.; Hörl A.; Rácz P.; Márton I.; Trügler A.; Krenn J. R.; Hohenester U. Ultrafast Strong-Field Photoemission from Plasmonic Nanoparticles. Nano Lett. 2013, 13 (2), 674–678. 10.1021/nl304365e. 23339740 PMC3573732

[ref13] Neacsu C. C.; Berweger S.; Olmon R. L.; Saraf L. V.; Ropers C.; Raschke M. B. Near-Field Localization in Plasmonic Superfocusing: A Nanoemitter on a Tip. Nano Lett. 2010, 10 (2), 592–596. 10.1021/nl903574a. 20067296

[ref14] Kubo A.; Pontius N.; Petek H. Femtosecond Microscopy of Surface Plasmon Polariton Wave Packet Evolution at the Silver/Vacuum Interface. Nano Lett. 2007, 7 (2), 470–475. 10.1021/nl0627846. 17298016

[ref15] Vogelsang J.; Robin J.; Nagy B. J.; Dombi P.; Rosenkranz D.; Schiek M.; Groß P.; Lienau C. Ultrafast Electron Emission from a Sharp Metal Nanotaper Driven by Adiabatic Nanofocusing of Surface Plasmons. Nano Lett. 2015, 15 (7), 4685–4691. 10.1021/acs.nanolett.5b01513. 26061633

[ref16] Gong Y.; Joly A. G.; Hu D.; El-Khoury P. Z.; Hess W. P. Ultrafast Imaging of Surface Plasmons Propagating on a Gold Surface. Nano Lett. 2015, 15 (5), 3472–3478. 10.1021/acs.nanolett.5b00803. 25844522

[ref17] Lemke C.; Leißner T.; Jauernik S.; Klick A.; Fiutowski J.; Kjelstrup-Hansen J.; Rubahn H.-G.; Bauer M. Mapping Surface Plasmon Polariton Propagation via Counter-Propagating Light Pulses. Opt. Express 2012, 20 (12), 12877 10.1364/OE.20.012877. 22714314

[ref18] Kahl P.; Podbiel D.; Schneider C.; Makris A.; Sindermann S.; Witt C.; Kilbane D.; Hoegen M. H.; Aeschlimann M.; Zu Heringdorf F. M. Direct Observation of Surface Plasmon Polariton Propagation and Interference by Time-Resolved Imaging in Normal-Incidence Two Photon Photoemission Microscopy. Plasmonics 2018, 13 (1), 239–246. 10.1007/s11468-017-0504-6.

[ref19] Kravtsov V.; Atkin J. M.; Raschke M. B. Group Delay and Dispersion in Adiabatic Plasmonic Nanofocusing. Opt. Lett. 2013, 38 (8), 1322 10.1364/OL.38.001322. 23595472

[ref20] Berweger S.; Atkin J. M.; Xu X. G.; Olmon R. L.; Raschke M. B. Femtosecond Nanofocusing with Full Optical Waveform Control. Nano Lett. 2011, 11 (10), 4309–4313. 10.1021/nl2023299. 21879749

[ref21] Yi J.-M.; Hou D.; Kollmann H.; Smirnov V.; Pápa Z.; Dombi P.; Silies M.; Lienau C. Probing Coherent Surface Plasmon Polariton Propagation Using Ultrabroadband Spectral Interferometry. ACS Photonics 2017, 4 (2), 347–354. 10.1021/acsphotonics.6b00821.

[ref22] Joly A. G.; Gong Y.; El-Khoury P. Z.; Hess W. P. Surface Plasmon-Based Pulse Splitter and Polarization Multiplexer. J. Phys. Chem. Lett. 2018, 9 (21), 6164–6168. 10.1021/acs.jpclett.8b02643. 30380891

[ref23] Crampton K. T.; Joly A. G.; El-Khoury P. Z. Direct Visualization of Counter-Propagating Surface Plasmons in Real Space-Time. J. Phys. Chem. Lett. 2019, 10 (19), 5694–5699. 10.1021/acs.jpclett.9b02151. 31498629

[ref24] Crampton K. T.; Joly A. G.; El-Khoury P. Z. Uncovering Surface Plasmon Optical Resonances in Nanohole Arrays through Interferometric Photoemission Electron Microscopy. Appl. Phys. Lett. 2022, 120 (8), 081102 10.1063/5.0082481.

[ref25] Wang B.; Aigouy L.; Bourhis E.; Gierak J.; Hugonin J. P.; Lalanne P. Efficient Generation of Surface Plasmon by Single-Nanoslit Illumination under Highly Oblique Incidence. Appl. Phys. Lett. 2009, 94 (1), 011114 10.1063/1.3068747.

[ref26] Ramanandan G. K. P.; Ramakrishnan G.; Kumar N.; Adam A. J. L.; Planken P. C. M. Emission of Terahertz Pulses from Nanostructured Metal Surfaces. J. Phys. D: Appl. Phys. 2014, 47 (37), 374003 10.1088/0022-3727/47/37/374003.

[ref27] Raether H. Surface Plasmons on Smooth and Rough Surfaces and on Gratings; Springer: Berlin/Heidelberg, 1988; 10.1007/BFb0048317.

[ref28] Kahl P.; Wall S.; Witt C.; Schneider C.; Bayer D.; Fischer A.; Melchior P.; Horn-von Hoegen M.; Aeschlimann M.; Meyer Zu Heringdorf F.-J. Normal-Incidence Photoemission Electron Microscopy (NI-PEEM) for Imaging Surface Plasmon Polaritons. Plasmonics 2014, 9 (6), 1401–1407. 10.1007/s11468-014-9756-6.

[ref29] Buckanie N. M.; Kirschbaum P.; Sindermann S.; Heringdorf F.-J. M. Z. Interaction of Light and Surface Plasmon Polaritons in Ag Islands Studied by Nonlinear Photoemission Microscopy. Ultramicroscopy 2013, 130, 49–53. 10.1016/j.ultramic.2013.03.007. 23688599

[ref30] Trebino R. Frequency-Resolved Optical Gating: The Measurement of Ultrashort Laser Pulses; Springer: Boston, MA, 2000; 10.1007/978-1-4615-1181-6.

[ref31] Trasatti S. Operative (Electrochemical) Work Function of Gold. Journal of Electroanalytical Chemistry and Interfacial Electrochemistry 1974, 54 (1), 19–24. 10.1016/S0022-0728(74)80376-1.

[ref32] Johnson P. B.; Christy R. W. Optical Constants of the Noble Metals. Phys. Rev. B 1972, 6 (12), 4370–4379. 10.1103/PhysRevB.6.4370.

[ref33] Lemke C.; Leißner T.; Klick A.; Fiutowski J.; Radke J. W.; Thomaschewski M.; Kjelstrup-Hansen J.; Rubahn H.-G.; Bauer M. The Complex Dispersion Relation of Surface Plasmon Polaritons at Gold/Para-Hexaphenylene Interfaces. Appl. Phys. B: Laser Opt. 2014, 116 (3), 585–591. 10.1007/s00340-013-5737-2.

[ref34] Zhang L.; Kubo A.; Wang L.; Petek H.; Seideman T. Imaging of Surface Plasmon Polariton Fields Excited at a Nanometer-Scale Slit. Phys. Rev. B 2011, 84 (24), 245442 10.1103/PhysRevB.84.245442.

[ref35] Zhang L.; Kubo A.; Wang L.; Petek H.; Seideman T. Universal Aspects of Ultrafast Optical Pulse Scattering by a Nanoscale Asperity. J. Phys. Chem. C 2013, 117 (36), 18648–18652. 10.1021/jp4076614.

[ref36] Wirth A.; Hassan M. Th.; Grguraš I.; Gagnon J.; Moulet A.; Luu T. T.; Pabst S.; Santra R.; Alahmed Z. A.; Azzeer A. M.; Yakovlev V. S.; Pervak V.; Krausz F.; Goulielmakis E. Synthesized Light Transients. Science 2011, 334 (6053), 195–200. 10.1126/science.1210268. 21903778

[ref37] Hassan M. Th.; Luu T. T.; Moulet A.; Raskazovskaya O.; Zhokhov P.; Garg M.; Karpowicz N.; Zheltikov A. M.; Pervak V.; Krausz F.; Goulielmakis E. Optical Attosecond Pulses and Tracking the Nonlinear Response of Bound Electrons. Nature 2016, 530 (7588), 66–70. 10.1038/nature16528. 26842055

[ref38] Escher M.; Weber N.; Merkel M.; Ziethen C.; Bernhard P.; Schönhense G.; Schmidt S.; Forster F.; Reinert F.; Krömker B.; Funnemann D. Nanoelectron Spectroscopy for Chemical Analysis: A Novel Energy Filter for Imaging x-Ray Photoemission Spectroscopy. J. Phys.: Condens. Matter 2005, 17 (16), S1329–S1338. 10.1088/0953-8984/17/16/004.

[ref39] Palik E. D.; Ghosh G. Handbook of Optical Constants of Solids; Academic Press: San Diego, 1998.

[ref40] Davidson A. C.; Hinkley D. V. Bootstrap Methods and Their Application; Cambridge Series in Statistical and Probabilistic Mathematics; Cambridge University Press: New York, NY, 1997.

