# Supplementary material for: Few-Cycle Surface Plasmon Polaritons
Source: Nano Lett. 2024 Feb 12;24(8):2637–42. doi: 10.1021/acs.nanolett.3c04991 (PMC10906073; doi:10.1021/acs.nanolett.3c04991)
Supplement: Supplementary file 1 — nl3c04991_si_001.pdf [file nl3c04991_si_001.pdf]

Supporting Information for

Few-Cycle Surface Plasmon Polaritons

Kazma Komatsu,[1] Zsuzsanna Pápa,[2,3] Thomas Jauk,[1] Felix Bernecker,[1] Lázár Tóth,[3] Florian Lackner,[1] Wolfgang E. Ernst,[1] Harald Ditlbacher,[4] Joachim R. Krenn,[4] Marcus Ossiander,[1,5] Péter Dombi,[2,3] and Martin Schultze[1]**

[1]Institute of Experimental Physics, Graz University of Technology, 8010 Graz, Austria.
[2]Wigner Research Centre for Physics, 1121 Budapest, Hungary.
[3]ELI-ALPS Research Institute, 6728 Szeged, Hungary.
[4]Institute of Physics, University of Graz, 8010 Graz, Austria.
[5]Harvard John A. Paulson School of Engineering and Applied Sciences, Harvard University, Cambridge, Massachusetts 02138, USA.

*Email: kazma.komatsu@tugraz.at, schultze@tugraz.at

1. Photoemission yield as a function of the laser intensity

Figure S1 displays the photoemission yield versus the laser intensity incident. The slope indicates a three-photon photoemission process.

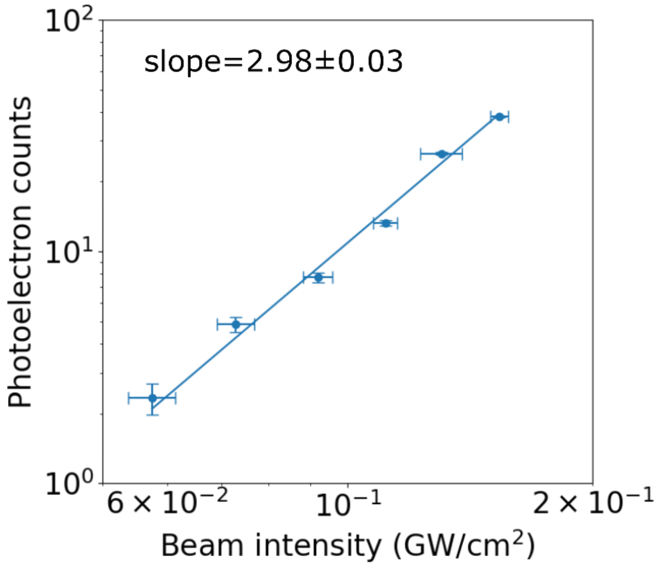

Figure S1. Photoemission yield as a function of laser intensity. Fitting the experimental data points yields a slope of approximately three, indicating a three-photon photoemission process.

2. Grating Coupler Modeling

As a first step, we compute the coupling efficiency for grating couplers with varying numbers of grooves. While the coupling efficiency increases with the number of grooves in both propagation directions, the bandwidth decreases (see Figure S2). From these results, we conclude that five grooves is a good compromise between a reasonable efficiency and bandwidth.

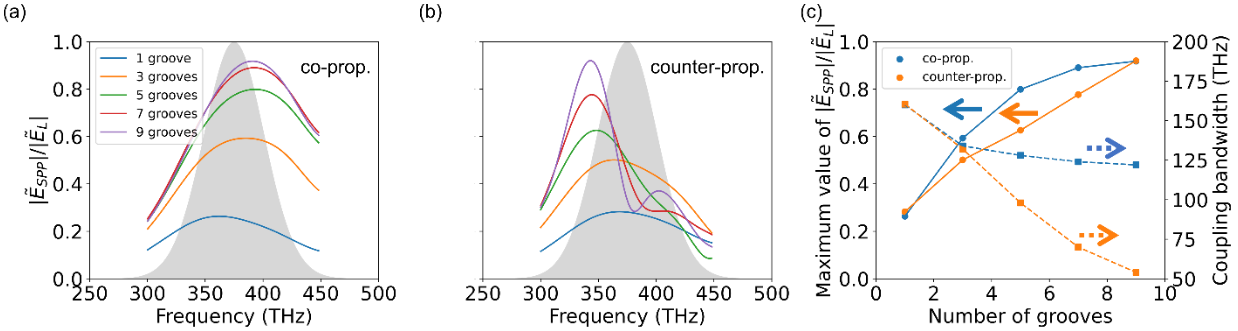

Figure S2. Coupling efficiency computed by FDTD simulations for gratings with a different number of grooves. (a) Numerical coupling efficiency of the co-propagating SPP and (b) of the counter-propagating SPP launched by a grating coupler. The coupling efficiency was estimated by $\frac{|\tilde{E}_{SPP}(\omega)|}{|\tilde{E}_L(\omega)|}$, where $\tilde{E}_{SPP}(\omega)$ and $\tilde{E}_L(\omega)$ are the spectral amplitude of the SPP field and the laser field. The laser spectrum is represented by the gray area for reference. (c) Maximum coupling efficiency (solid lines) and coupling bandwidth (dashed lines) as a function of groove number.

We then optimize the duty cycle of the grating coupler. Figure S3 shows the coupling efficiencies for different duty cycles. A duty cycle of 50 % already maximizes the coupling efficiency of co-propagating SPPs and also provides a good coupling efficiency for counter-propagating SPPs. The coupling bandwidth is independent of the duty cycle.

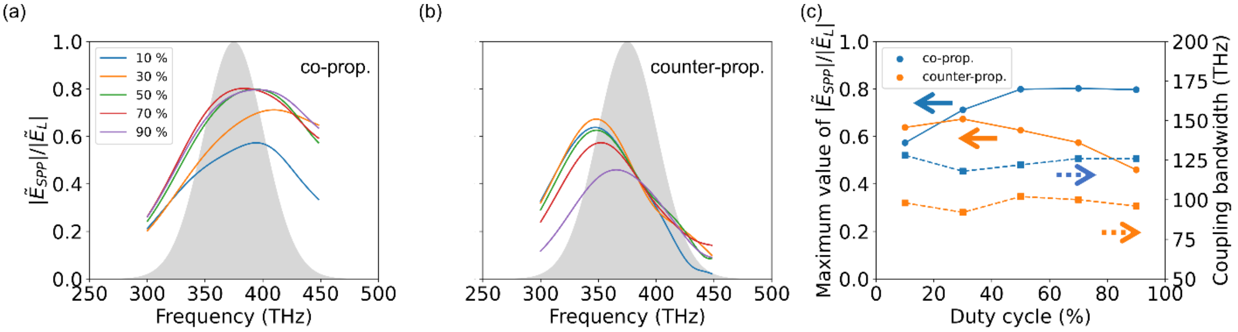

Figure S3. Coupling efficiency for gratings with different duty cycle. (a) Numerical coupling efficiency of the co-propagating SPP and (b) of the counter-propagating SPP. The number of grooves is fixed to five in both cases. The laser spectrum is represented by the gray area for reference. (c) Dependence of the maximum coupling efficiency (solid lines) and coupling bandwidth on the duty cycle (dashed lines).

As the last step, we calculate the dependence of the coupling efficiency on the grating constant (Figure S4). Thereby, a grating period of 390 nm proves to be a formidable candidate, as it provides reasonable coupling efficiency for both co- and counter-propagating SPP wavepackets. A grating period of 340 nm could be a suitable alternative, facilitating the coupling of a similar bandwidth of both co- and counter-propagating SPP wavepackets. However, this comes at the expense of considerably reduced efficiency for the counter-propagating SPP.

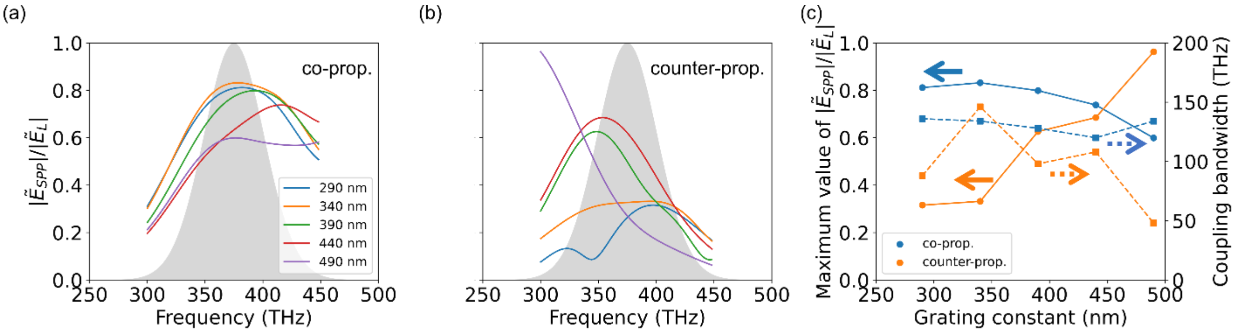

Figure S4. Coupling efficiency computed by FDTD simulations for gratings with various grating constants. (a) Numerical coupling efficiency of the co-propagating SPP and (b) of the counter-propagating SPP. The number of grooves is fixed to five with a duty cycle of 50%. The laser spectrum is represented by the gray area for reference. (c) Maximum coupling efficiency (solid lines) and coupling bandwidth as a function of the grating constant (dashed lines).

3. Pulse width retrieval from the photoemission autocorrelation traces

To obtain the temporal durations of the SPP wavepackets, we initially extract the temporal width of the laser pulse from the autocorrelation trace $I_{AC}(\tau)$ by using the third-order autocorrelation formula [S1]

$$I_{AC}(\tau) = \int {I_L}^2(t) I_L(t-\tau) dt \propto \int e^{-\frac{2t^2}{2\sigma_L^2}} e^{-\frac{(t-\tau)^2}{2\sigma_L^2}} dt \propto e^{-\frac{\tau^2}{2\left(\frac{3}{2}\sigma_L^2\right)}}. \tag{S1}$$

The laser intensity $I_L(t)$ was assumed to have a Gaussian envelope. The width of the autocorrelation σ_{AC} is linked to the laser's width σ_L by

$$\sigma_{\mathrm{AC}} = \sqrt{\frac{3}{2}} \sigma_L. \tag{S2}$$

The extracted temporal width σ_L was then used in the third-order cross correlation formula in order to retrieve the durations of the SPP σ_{SPP}, by fitting the experimental cross correlation traces.

4. SPP spectrum retrieval from the experimental data

According to Ref. [S1], and because $I_{SPP}^2 < 0.1 I_L^2$,we can express the autocorrelation $I_{AC}(\tau)$ as Eq. (S1) and the third-order cross correlation as

$$I_{CC}(\tau) = \int {I_L}^2(t) I_{SPP}(t-\tau) dt\,. \tag{S3}$$

Using a convolution approach, the Fourier transforms of $I_{AC}(\tau)$ and $I_{CC}(\tau)$ are written by

$$\tilde{I}_{AC}(\omega) = \mathrm{FT}[I_{AC}(t)] \propto \mathrm{FT}\left[{I_L}^2(t)\right] \mathrm{FT}[I_L(t)], \tag{S4}$$

and

$$\tilde{I}_{CC}(\omega) = \mathrm{FT}[I_{CC}(t)] \propto \mathrm{FT}\left[{I_L}^2(t)\right] \mathrm{FT}[I_{SPP}(t)]. \tag{S5}$$

Thus, the SPP spectrum $\tilde{I}_{SPP}(\omega)$ is governed by

$$\tilde{I}_{SPP}(\omega) = \mathrm{FT}[I_{SPP}(t)] \propto \frac{\tilde{I}_{CC}(\omega)}{\tilde{I}_{AC}(\omega)} \mathrm{FT}[I_L(t)]. \tag{S6}$$

$\mathrm{FT}[I_L(t)]$ is given by the laser spectrum.

5. Estimation of the field coupling efficiency from the correlation traces

The field coupling efficiency is obtained through the ratio of the intensity of the cross-correlation to that of the autocorrelation. Using Eq. (S4) and Eq. (S5), the field coupling efficiency $\eta^{field}(\omega)$ can be expressed as follows

$$\eta^{field}(\omega) = \frac{|\tilde{E}_{SPP}(\omega)|}{|\tilde{E}_L(\omega)|} = \frac{\sqrt{\tilde{I}_{SPP}(\omega)}}{\sqrt{\tilde{I}_L(\omega)}} = \frac{\sqrt{\mathrm{FT}[I_{SPP}(t)]}}{\sqrt{\mathrm{FT}[I_L(t)]}} = \frac{\sqrt{\tilde{I}_{CC}(\omega)}}{\sqrt{\tilde{I}_{AC}(\omega)}}. \qquad \text{(S7)}$$

6. Evaluation of the laser-plasmon phase

From the FDTD simulations, the phase of the light pulse and the SPP wavepackets at a traveling distance x and a frequency ω is given by

$$\phi_{SPP/laser}(x,\omega) = \arctan\left(\frac{\mathrm{Im}\left(\mathrm{FT}\left[E_{SPP/laser}(x,t)\right]\right)}{\mathrm{Re}\left(\mathrm{FT}\left[E_{SPP/laser}(x,t)\right]\right)}\right), \qquad \text{(S8)}$$

where $E_{SPP/laser}(x,t)$ denotes the field amplitude of the SPP (light). The phase φ as a function of x follows

$$\varphi_{SPP/laser}(x) = \frac{\int \phi_{SPP/laser}(x,\omega)\cdot I_{SPP/laser}(x,\omega)d\omega}{\int I_{SPP/laser}(x,\omega)d\omega}, \qquad \text{(S9)}$$

where $I_{SPP/laser}(x,\omega)$ represents the magnitude of $\mathrm{FT}\left[E_{SPP/laser}(x,t)\right]$. The laser-plasmon phase $\varphi_{l-p}(x)$ is then given by $\varphi_{l-p}(x) = \varphi_{SPP}(x) - \varphi_{laser}(x)$.

Experimentally, we firstly fit the envelope of the cross-correlation traces with the Gaussian. Then, the laser-plasmon phase $\varphi_{l-p}^{exp}(x)$ at a traveling distance x is obtained by

$$\varphi_{l-p}^{exp}(x) = 2\pi\cdot\frac{t_{car}(x) - t_{env}(x)}{T_{CC}}. \qquad \text{(S10)}$$

Here $t_{env}(x)$ is the center position of the fitted envelope and $t_{car}(x)$ is the time when the cross-correlation yield gets maximum at the distance x. T_{CC} represents the period of the cross-correlation traces.

7. Analytical calculation of the laser-plasmon phase

For an analytical approach, we retrieve the center frequency of the SPP waves from the FDTD simulations and calculated the group and phase velocity. Subsequently, the analytical phase is given by

$$\varphi^{\text{ana.}}(x) = \frac{2\pi}{T} \cdot \left(\frac{x}{v_{ph}} - \frac{x}{v_g} \right), \tag{S11}$$

where x is the traveling distance, v_{ph} (v_g) the phase (group) velocity, and T the period of the SPP or light waves. To obtain the offset in the laser-plasmon phase using the analytical solution $\varphi_{l-p}^{\text{ana.}}(x) = \varphi_{\text{SPP}}^{\text{ana.}}(x) - \varphi_{\text{laser}}^{\text{ana.}}(x)$, we apply a fitting routine to the FDTD results and the offset as free parameter.

References

(S1) Trebino, R. *Frequency-Resolved Optical Gating: The Measurement of Ultrashort Laser Pulses*; Springer US: Boston, MA, 2000. https://doi.org/10.1007/978-1-4615-1181-6.
